# Anti-inflammatory recombinant TSG-6 stabilizes the progression of focal retinal degeneration in a murine model

**DOI:** 10.1186/1742-2094-9-59

**Published:** 2012-03-27

**Authors:** Jingsheng Tuo, Xiaoguang Cao, Defen Shen, Yujuan Wang, Jun Zhang, Joo Youn Oh, Darwin J Prockop, Chi-Chao Chan

**Affiliations:** 1 Laboratory of Immunology, National Eye Institute, National Institutes of Health, Bethesda, MD, USA; 2 Institute for Regenerative Medicine, Texas A&M Health Science Center, College of Medicine at Scott & White, Temple, TX, USA; 3 Laboratory of Immunology, National Eye Institute, National Institutes of Health, 10 Center Drive, Building 10, Room 10 N103, NIH/NEI, Bethesda, MD 20892-1857, USA

**Keywords:** Age-related macular degeneration, Animal model, IL-17a, TNF-α, TSG-6, Treatment

## Abstract

**Background:**

Inflammatory responses are detected in the retina of patients with age-related macular degeneration and *Ccl2^-/-^/Cx3cr1^-/- ^*mice on rd8 background,(*Ccl2^-/-^/Cx3cr1^-/- ^*mice) a model that develops progressive age-related macular degeneration-like retinal lesions including focal photoreceptor degeneration, abnormal retinal pigment epithelium and A2E accumulation. Tumor necrosis factor-inducible gene 6 protein is an anti-inflammatory protein and has been shown to improve myocardial infarction outcome and chemically injured cornea in mice by suppressing inflammation. In this study, we evaluated the effect of an intravitreous injection of recombinant TSG-6 on the retinal lesions of *Ccl2^-/-^/Cx3cr1^-/- ^*mice.

**Methods:**

Recombinant TSG-6 (400 ng) was administered by intravitreous injection into the right eye of six-week-old C*cl2^-/-^/Cx3cr1^-/- ^*mice. Their left eye was injected with phosphate-buffered saline as a control. Funduscopic pictures were taken before injection and sequentially once a month after injection. The mice were killed two months after injection and the ocular histology examined. Retinal A2E, a major component of lipofuscin, was measured by high performance liquid chromatography. The microarray of ocular mRNA of 92 immunological genes was performed. The genes showing differentiated expression in microarray were further compared between the injected right eye and the contralateral (control) eye by [real-time quantitative reverse transcription polymerase chain reaction] qRT-PCR.

**Results:**

The continuous monitoring of the fundus for two months showed a slower progression or alleviation of retinal lesions in the treated right eyes as compared with the untreated left eyes. Among 23 pairs of eyes, the lesion levels improved in 78.3%, stayed the same in 8.7% and progressed in 13.0%. Histology confirmed the clinical observation. Even though there was no difference in the level of A2E between the treated and the untreated eyes, microarray analysis of 92 immune genes showed that *IL-17a *was substantially decreased after the treatment. Expression of *TNF-α *showed a similar pattern to *IL-17a*. The results were consistent in duplicated arrays and confirmed by qRT-PCR.

**Conclusions:**

We concluded that intravitreous administration of recombinant TSG-6 might stabilize retinal lesions in *Ccl2^-/-^/Cx3cr1^-/- ^*mice on rd8 background. Modulation of ocular immunological gene expressions, especially IL-17a, could be one of the mechanisms.

## Background

Age-related macular degeneration (AMD) is a common cause of irreversible central blindness in elderly patients worldwide [1]. The pathological features of this neurodegenerative disease include degeneration and atrophy of the photoreceptors and the retinal pigment epithelium (RPE) in the macula. The advanced stage of AMD presents as the exudative/neovascular or 'wet' form with choroidal neovascularization, and the geographic atrophy or 'dry' form with loss of the photoreceptors and RPE. Except for the suppression of choroidal neovascularization, there are few options for AMD intervention. Care for intermediate AMD and dry AMD is limited to risk factor management. Smoking cessation, body mass reduction, and specific vitamins and nutrient supplements have been reported to slow disease progression [2].

We have previously reported that genetically engineered *Ccl2^-/-^/Cx3cr1^-/- ^*mice on rd8 background (*Ccl2^-/-^/Cx3cr1^-/-^*) developed a broad spectrum of AMD-like pathology with early onset and high penetrance [3-5]. The retinal abnormalities of this strain include focal deep retinal lesions, photoreceptor disorganization and degeneration, retinal pigment epithelial degeneration and atrophy, A2E accumulation, and some with choroidal neovascularization. The spontaneous retinal lesions are generally symmetric in both eyes and become worse with age. We have successfully demonstrated the beneficial effects of long-term dietary intake of long-chain omega-3 polyunsaturated fatty acids (n-3) to alleviate the retinal lesions of *Ccl2^-/-^/Cx3cr1^-/- ^*mice. Chow rich in n-3 was able to decelerate the retina lesions observed by funduscopy, reserve the retinal structure observed by histology and reduce the A2E level (retina autofluorescence component) measured by HPLC [6]. We also reported that the subretinal injection of an adeno-associated virus vector in which a portion of the soluble vascular endothelial growth factor (VEGF) receptor gene was cloned to trap excess VEGF-A either stabilizes or arrests the progression of retinal lesions in *Ccl2^-/-^/Cx3cr1^-/- ^*mice [7]. This strain was on rd8 background (unpublished data), but exhibited 100% penetrance and more prominent focal lesions at an earlier age compared with the rd8 with a single base deletion in the *Crb1 *gene [8]. Due to the multiple functions of a gene and disturbance of the whole pathway when key genes are disrupted, the loss of *Ccl2 *and *Cx3cr1 *on rd8 background could cause the malfunction of pathways other than the chemokine and chemokine receptor. This is probably why a broad spectrum of pathological features was observed in the inbred strain. We have characterized the abnormalities in macrophage immunology, oxidative stress and chaperone chemistry in the retinal tissue of *Ccl2^-/-^/Cx3cr1^-/- ^*mice [3,4,6,7,9-17]. The evidence collected from previous studies on this mouse model has encouraged us to test other potential options for AMD therapy.

Inflammation, among other factors, has been suggested to play an important role in AMD pathogenesis. Elevated inflammatory mediators have been found in the retinal tissues of AMD patients and *Ccl2^-/-^/Cx3cr1^-/- ^*mice [6,7,9,11,13]. Tumor necrosis factor-inducible gene 6 protein (TSG-6) was found to be anti-inflammatory and has been shown to improve myocardial infarction outcome and chemically injured cornea in mice by reducing inflammation in the heart and cornea, respectively [18-23]. In this study, we explored the potential beneficial effect of intravitreal administration of TSG-6 on the retinal lesion of *Ccl2^-/-^/Cx3cr1^-/- ^*mice. Funduscopy, histology, the level of retinal lipofuscin and determination of immune response-related molecules were used to evaluate the effects of the intervention.

## Methods

### Animals and treatment

*Ccl2^-/-^/Cx3cr1^-/- ^*mice on rd8 background and wild type control (C57BL/6) were bred in-house. The study was conducted in compliance with the ARVO statement for the use of animals, and all animal experiments were performed under protocols approved by the Institutional Animal Care and Use Committee of National Eye Institute, National Institutes of Health, USA.

A one-time intravitreous injection of 400 ng of recombinant TSG-6 (R&D Systems, (Minneapolis, MN, USA), Cat. No. 2326-TS) in 1 μL volume was performed into the right eye of six-week-old *Ccl2^-/-^/Cx3cr1^-/- ^*mice on rd8 background (n = 31). Their left eye was administered 1 μL phosphate-buffered saline by intravitreous injection and served as the control. Eyes were harvested two months after the injection for various measurements. The retina of C57BL/6 was used for gene expression assay control.

### Fundus photography

A Karl Storz veterinary otoendoscope (Karl Storz, Tuttlingen, Germany) coupled with a Nikon D90 digital camera was used for taking mouse fundus photographs before the injection and every month after the injection for two months [24]. The fundus photograph was taken after pupil dilation (1% tropicamide ophthalmic solution; Alcon Inc., Fort Worth, TX, USA) and an intraperitoneal injection of ketamine (1.4 mg/mouse) and xylazine (0.12 mg/mouse) was used for systemic anesthesia. We evaluated the lesion changes by comparing sequential photographs taken in the same fundus area of each eye. Progression was defined as a > 10% increase in the number of retinal lesions (Grade +1), a > 50% increase in the lesion size in at least one out of three of the lesions (Grade +2), more than five fused lesions or the appearance of more than two chorioretinal scars (Grade +3), or diffuse chorioretinal scars (Grade +4) in comparison with the previous observation. Regression was defined as a > 10% decrease in the number of retinal lesions (Grade -1), a > 50% decrease in lesion size in at least one out of three of the lesions (Grade -2), a > 50% disappearance of retinal lesions (Grade -3) or a total disappearance of retinal lesions. To avoid bias, evaluation of the pictures was conducted by a masked observer. The lesion scores of each eye were estimated by cross-comparison of the same area based on above criteria.

### Histology

Whole eyes were fixed in 4% glutaraldehyde-10% formalin and then embedded in methacrylate. The eyes were serially sectioned in the vertical pupillary-optic nerve plane. Each eye was cut into six sections. All sections containing the entire eye were stained with hematoxylin and eosin. If an ocular lesion was observed, another six to twelve sections were cut through the lesion. These slides were stained with Periodic Acid Schiff stain to highlight Bruch's membrane and the basement membrane of small neovascular vessels.

### Transmission electron microscopy

The whole eyes were fixed in 2% glutaraldehyde and 2% paraformaldehyde. The tissue was embedded in Durcupan epoxy resin. Six 1 μm-thick sections stained with toluidine blue were examined under light microscopy. Based on the lesions shown on the thick sections, ultrathin sections of these lesions were taken and stained with uranyl acetate and lead citrate for examination under a JEOL1010 electron microscope (JEOL USA, Inc., Peabody, MA, USA). Four eyes from two mice were used for transmission electron microscopy.

### Retinal lipofuscin extraction and quantification

A2E ([2,6-dimethyl-8-(2,6,6-trimethyl-1-cyclohexen-1-yl)-1E,3E,5E,7E-octatetra-enyl]-1-(2-hydroxyethyl)-4-[4-methyl-6(2,6,6-trimethyl-1-cyclohexen-1-yl) 1E,3E,5E,7E-hexatrienyl]-pyridinium) is the major component of lipofuscin fluorophores generated from the visual cycle flux of all-trans-retinal. The molecule is particularly relevant to aging and AMD pathogenesis [25]. The mice were kept in the dark for > 12 hours before they were killed. The whole eyes were removed in a dark room under dim red light and homogenized. A2E was extracted with chloroform and methanol as previously described [26]. The extracts dissolved in methanol were separated by HPLC (Agilent 1100 LC, Wilmington, DE, USA) and detected by an UV detector at a wavelength of 435 nm. A gradient of 40% to 95% acetonitrile and H_2_O in 0.1% trifluoracetic acid was used to elute A2E on a reverse-phase C18 column (Agilent, eclipse XD8-C18, 5 μm, 4.6 × 150 mm) at a flow-rate of 1.0 mL/min. A2E was quantified using external A2E standards [27].

### cDNA synthesis

Total RNA from mouse retina and RPE was extracted using Trizol (Invitrogen, Carlsbad, CA, USA). cDNA was synthesized using 10 μg total RNA in a total volume of 100 μL using reverse transcriptase (TaqMan reverse transcriptase reagents, Applied Biosystems, Foster City, CA, USA).

### TaqMan gene expression array

The array to identify altered immune response gene expression after TSG-6 treatment was performed using a TaqMan Array Mouse Immune Response 96-well Plate according to the manufacturer's protocols (Applied Biosystems). The plate contained 92 assays to immune response-associated genes and four assays to candidate endogenous control genes. The genes are listed in Additional file 1: Table S1. The cDNA (100 μL) was mixed with H_2_O and 2X TaqMan Universal PCR Mix (Applied Biosystems) to make 2,500 μL of master mix that was evenly loaded into each well (25 μL/well). The reaction was run on an ABI 7500 System (Applied Biosystems). Expression values were determined with DataAssist v2.0 Software (Applied Biosystems).

### Quantification of gene expression by [real-time quantitative reverse transcription polymerase chain reaction] qRT-PCR

The synthesis of the cDNA was described above. The primers and probe for *IL-17a *and *TNF*-*α *were purchased from Applied Biosystems as inventoried TaqMan gene expression reagents. Relative qRT-PCR was performed according to manufacturer instructions. To determine the cycle threshold (Ct) values, the threshold level of fluorescence was set manually in the early phase of PCR amplification. ABI SDS 1.3.1 software and the 2^-ΔΔCt ^analysis method were used to determine relative amounts of product using *GAPDH *as an endogenous control. The relative expression was normalized first by the level of *GAPDH *from the same cDNA sample and again normalized to the transcript level in untreated eyes due to treatment. Each sample was analyzed in triplicate.

### Statistical analysis

The rates of progression and regression between groups were compared by the Chi-square test. Multiple means were compared by paired *T*-test. Differences were considered significant when *P *< 0.05.

## Results

Two independent experiments (n = 22 in the first experiment, n = 9 in the second experiment) were performed and yielded repeatable results.

### TSG-6 arrested retinal lesions

**Figure 1:**
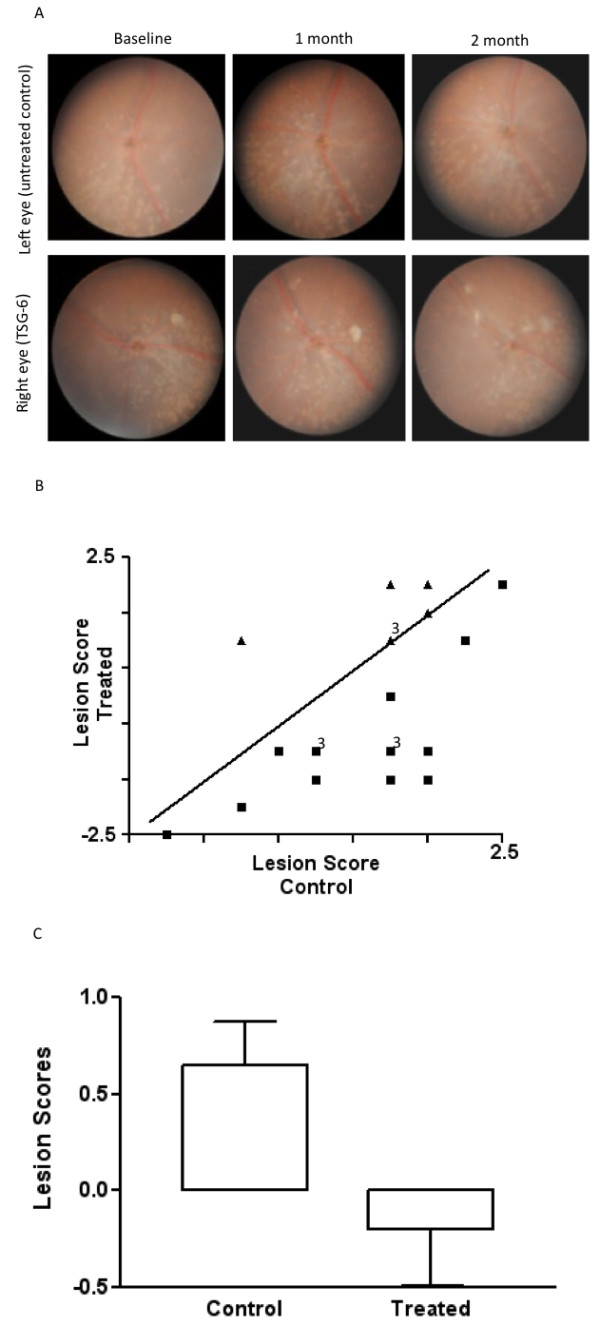
**Continuous funduscopic monitoring**. **(A) **Representative funduscopic photographs of a pair of eyes in one mouse monitored for two months. The TSG-6 treated eye (right) showed fewer retinal lesions than the control eye (left) after two months. **(B) **Pairwise plotting of 23 pairs of eyes showing different progressing scores between the two eyes. Some eye pairs had identical scores and the numbers of overlapped dots are listed by the dots. The triangles indicate stable or worse lesions. The squares indicate improved lesions. **(C) **The average scores of retinal lesions in treated and untreated eyes showing lower scores in the treated eyes.

The right eyes that received TSG-6 showed retinal lesion arrests as compared with the contralateral left eyes (control), in which the retinal lesions progressed two months after injection (Figure 1A). The plot of 23 pairs of eyes showed that 16 treated eyes were healthier, four treated eyes remained at the same lesion scales and three treated eyes became worse (increased size and number of the lesions) relative to the contralateral eye by funduscopic examination two months after injection (Figure 1B). The natural course of the retinal lesions in *Ccl2^-/-^/Cx3cr1^-/- ^*mice is to worsen with time [4]; therefore, most untreated left eyes showed worsened lesions. The average lesion score changes showed improvement (decreased size and number of the lesions) in the treated right eyes compared with the control left eyes (*P *< 0.01; Figure 1C).

**Figure 2:**
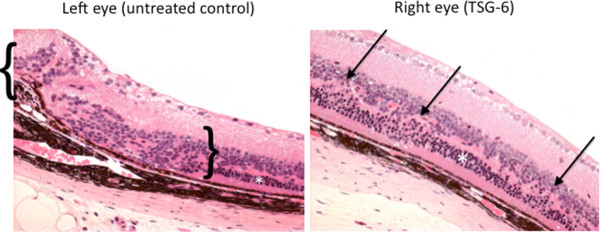
**Histological examination of the eyes**. Representative photomicrographs of the retinas from a pair of eyes from one mouse: healthier retinal structure was indicated by less photoreceptor cell degeneration (white asterisks), fewer retinal lesions (arrows) and a thicker retinal outer layer in the treated eye (right) in comparison to the untreated eye (left), which shows a larger area (blankets) with loss of photoreceptor cells two months after TSG-6 injection. (Hematoxylin & eosin, scale bar = 60 μm).

**Figure 3:**
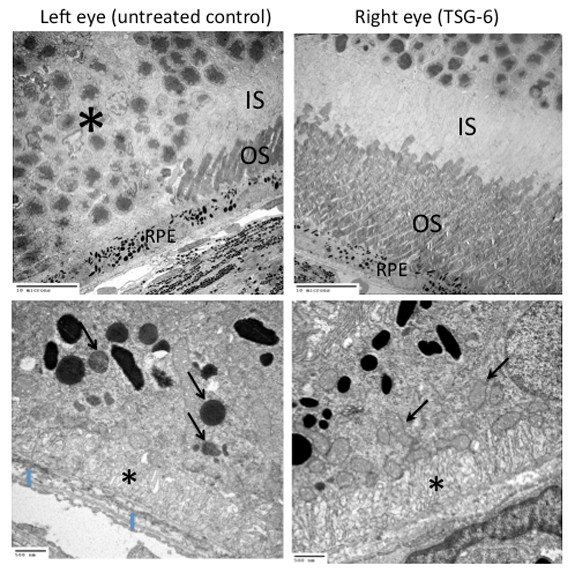
**Transmission electron micrographs of the retina**. Left upper panel: the retina of the untreated eye shows degeneration of RPE and photoreceptors. The photoreceptors are dystrophic with many dislocated nuclei (asterisk) and loss of both inner and outer segments. Left lower panel: the RPE cell contains lipofuscin-like liposomal inclusions (arrows) and decreased basal infoldings (asterisk). Bruch's membrane shows electron dense debris (blue arrows). Right upper panel: the ultrastructure of the retina from a treated eye is intact with well-preserved photoreceptors including healthy inner (IS) and outer (OS) segments and RPE cells. Right lower panel: the RPE cell contains many normal mitochondria (arrows) and basal infoldings (asterisk).

The histological observations indicated better retinal morphology in the treated eyes than in the non-treated eyes, characterized by less photoreceptor atrophy, smaller and fewer retinal lesions and thicker retinal outer layers (Figure 2). The histological examination between the right and left eyes from 10 pairs of eyes revealed a decreased lesion severity in six pairs, similar lesion severity in two pairs and an increased lesion severity in only two pairs two months after injection. The improvement in the eyes with decreased lesion severity identified on routine histology was confirmed by transmission electron microscopy. The retinal ultrastructure illustrated better preserved photoreceptor layers in the treated eyes compared with the untreated eyes. The retina of the untreated eyes showed degeneration of RPE cells, photoreceptors and Bruch's membrane (Figure 3).

### TSG-6 intervention did not alter the accumulation of lipofuscin in the retina

**Figure 4:**
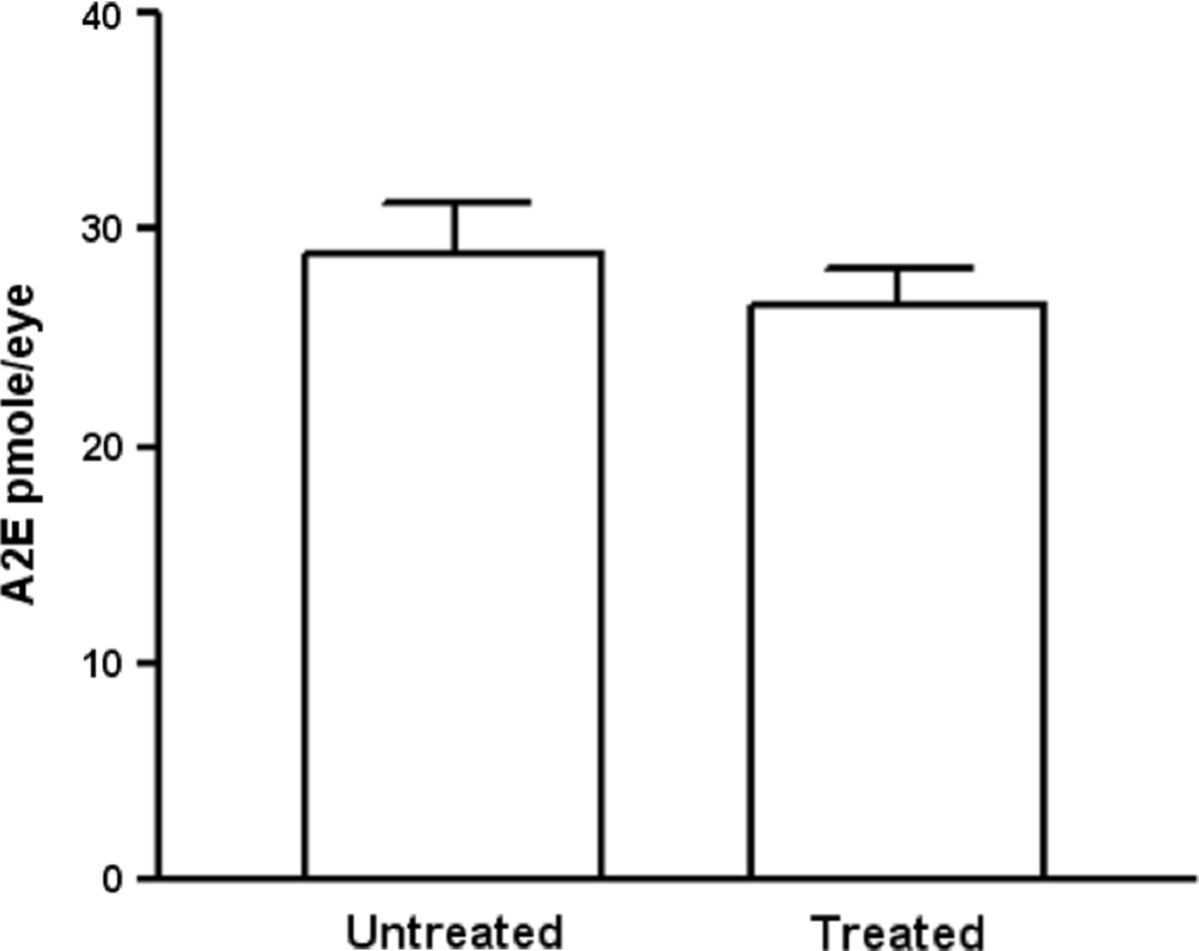
**Quantification of retinal A2E**. There was no difference of A2E levels in the retina between TSG-6 treated eyes (n = 7) and the untreated eyes (n = 7) two months after treatment.

There was no statistical difference in the amount of A2E between the treated eyes and the untreated eyes of *Ccl2^-/-^/Cx3cr1^-/- ^*mice (Figure 4).

### TSG-6 intervention substantially decreased *IL-17a *and *TNF-α *expression

**Figure 5:**
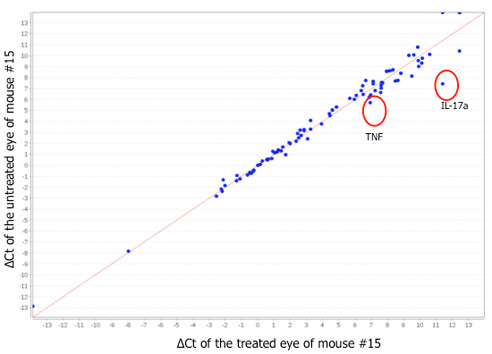
**Gene expression array in the retina**. Pair plot of 92-gene expression of a pair of treated and untreated eyes (mouse #15). The expression of the majority of immune response genes does not differ substantially between the pair, as shown in the distance from the bisecting line. However, expression of *IL-17a *was substantially reduced in the treated eye compared with the control eye. A duplicated array of another pair of eyes showed the same pattern. The *TNF-α *expression was also consistently reduced in the duplicated array of the treated eye compared with the control eye.

**Figure 6:**
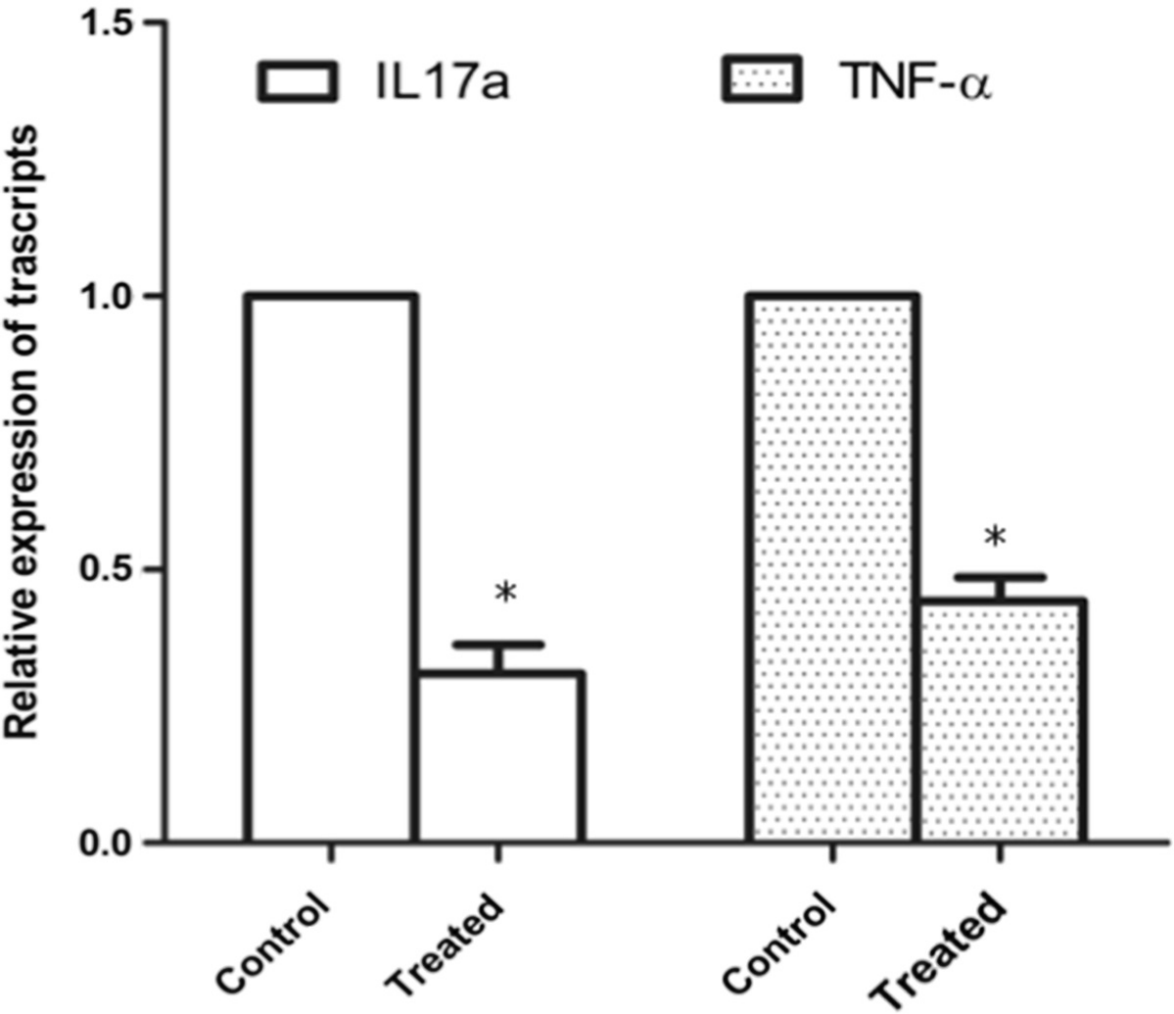
**qRT-PCR of *IL-17a *and *TNF-α***. Significantly lower expression of *IL-17a *and *TNF-α *transcripts can be seen in the eyes treated with TSG-6 compared with the controls. *P < 0.05.

To study the role of TSG-6 on immune response in the *Ccl2^-/-^/Cx3cr1^-/- ^*retina, we performed a pathway mRNA array for immune response. Although the majority of these genes were suppressed after the treatment, *IL-17a *was consistently down-regulated by a substantial degree in the TSG-6 treated eyes (Figure 5). The quantitative real time PCR assay in an additional five pairs of eyes further confirmed a three-fold decrease of *IL-17a *in the retinal tissue after the treatment (Figure 6). Interestingly, *TNFα-α *expression showed a similar pattern to *IL-17a *expression (Figure 6).

## Discussion

We found that intravitreous delivery of recombinant TSG-6 protein could stabilize the progression of retinal lesions in *Ccl2^-/-^/Cx3cr1^-/- ^*mice on rd8 background, indicated by the better preserved morphology of retinal structure compared with untreated control eyes. The improvement of the degenerative lesion was correlated to the altered immune gene expression, particularly the decreased expression of *IL-17a *and *TNF-α*.

The pro-inflammatory cytokine tumor necrosis factor-alpha (TNF-α) is one of the primary mediators of the acute phase response. We previously reported an elevated expression of TNF-α in the retina of *Ccl2^-/-^/Cx3cr1^-/- ^*mice [6]. Of interest in the present study is that expression of the gene for TNF-α was reduced after the TSG-6 treatment, an observation that is consistent with the anti-inflammatory effects of the protein [18,20,21,23]. TNF-α and other pro-inflammatory cytokines induce synthesis of TSG-6 in many different cells, but TSG-6 creates a negative feedback loop that modulates the inflammatory response. The protein was previously shown to decrease inflammation by binding to pro-inflammatory fragments of hyaluronan, increasing the inhibitory activity of inter-α-inhibitor, and inhibiting migration of neutrophils [21,23,28]. More recently it was shown to modulate nuclear factor-κB signaling in resident macrophages by its interaction with CD44 [29]. The anti-inflammatory activity has been directly demonstrated in a number of rodent models of inflammation including models of arthritis [23], carrageenan- or IL-1-induced inflammation in an air pouch [30], myocardial infarction [19], chemical injury to the cornea [22] and peritonitis [29].

We have previously reported that inflammation and oxidative stress are major pathogenetic mechanisms inducing retinal lesions in *Ccl2^-/-^/Cx3cr1^-/- ^*mice [6,9,13]. In contrast to our previous intervention studies using this strain [6,7], we did not find an altered A2E level in this study. A2E, a major component of lipofuscin, is generated by a series of oxidative stress-mediated redox reactions [31] and the concentration of A2E is high in the retina of *Ccl2^-/-^/Cx3cr1^-/- ^*mice [4]. This might be evidence that the beneficial effects of TSG-6 administration come from anti-inflammatory effects other than the inhibition of oxidative stress.

A subset of effector helper T-cells, IL-17-producing T-cell (Th17), is largely implicated in the pathogenesis of various autoimmune diseases [32]. Autoimmunity has been cited in the pathogenesis of AMD [33-36]. Significantly increased levels of IL-22, a member of the Th17 family, and IL-17 were detected in the serum of AMD patients as compared with non-AMD controls [37]. We found that, out of 92 immune genes tested, *IL-17a *was significantly down-regulated after TSG-6 treatment. This suggests that TSG-6 has a role in the regulation of IL-17 that associates with the retinal lesion. Interestingly, IL-17 was shown to have additive effects with TNF-α and IL-1 on the up-regulation of TSG-6 expression in the synovium of patients with rheumatoid arthritis [38].

Our data cannot exclude mechanisms other than the anti-inflammatory role of TSG-6 in the outcome of the treatment. TSG-6 contains a hyaluronan-binding link domain and thus is a member of the hyaluronan-binding protein family, also called hyaladherins. In addition to its anti-inflammatory effects in binding fragments of hyaluronan, the hyaluronan-binding domain is known to be involved in extracellular matrix stability [39]. Interesting, a recent study reported that IL-17F, sharing high homology with other IL-17 family members including IL-17A, can regulate extracellular matrix stability and stimulate cartilage degradation by increasing the expression of collagenases (matrix metalloproteinase (MMP)-1 and -13) and stromelysin-1 (MMP-3) and by decreasing the expression of their inhibitors (tissue inhibitor of metalloproteinase-2 and -4), type II collagen, aggrecan and link protein in chondrocytes [40]. It is believed that defective biosynthesis and/or degradation of the extracellular matrix has the potential to alter the morphologic and functional characteristics of Bruch's membrane, an initial site of pathological change during AMD development. The aging of Bruch's membrane was associated with an exponential increase in the percentage of pro-MMPs bound to the membrane [41].

## Conclusion

The findings reported here demonstrate that intravitreous delivery of recombinant TSG-6 has a modest effect in delaying or improving the progression of retinal lesions in *Ccl2^-/-^/Cx3cr1^-/- ^*mice on rd8 background. Therefore, TSG-6 may prove to be a supplement to the beneficial effects in AMD treatment obtained by long-term dietary therapy with omega-3 polyunsaturated fatty acids and subretinal injection of adeno-associated virus encoding soluble VEGF receptor [6,7]. The TSG-6 appeared to act primarily by suppressing inflammation as indicated by its down-regulation of IL-17a and TNF-α.

## Abbreviations

A2E: ([2,6-dimethyl-8-(2,6,6-trimethyl-1-cyclohexen-1-yl)-1E,3E,5E,7E-octatetra-enyl]-1-(2-hydroxyethyl)-4-[4-methyl-6(2,6,6-trimethyl-1-cyclohexen-1-yl) 1E,3E,5E,7E-hexatrienyl]-pyridinium); AMD: Age-related macular degeneration; Ct: Threshold cycle; HPLC: High performance liquid chromatography; IL: Interleukin; MMP: Matrix metalloproteinase; PCR: Polymerase chain reaction; RPE: Retinal pigment epithelium; TNF: Tumor necrosis factor; Th17: IL-17-producing T-cell; TSG-6: Tumor necrosis factor-inducible gene 6 protein; VEGF: Vascular endothelial growth factor

## Competing interests

The authors declare that they have no competing interests.

## Authors' contributions

JT designed the study, conducted molecular assays and the data analysis, and drafted the manuscript. XC, DF and YW carried out the clinical and pathological observation and maintained the records. JZ performed the electron microscope study. JYO and DJP participated in the study design and manuscript revision. CCC initiated and designed the study, analyzed data, graded clinical and pathological scores and critically reviewed the manuscript. All authors read and approved the final manuscript.

## Supplementary Material

Additional file 1 **Table S1**. The list of mouse immune response genes. Click here for file

## References

[B1] Gehrs KM Anderson DH Johnson LV Hageman GS Age-related macular degeneration-emerging pathogenetic and therapeutic concepts Ann Med 2006 38 450 471 10.1080/07853890600946724 17101537 PMC4853957

[B2] Smith TC Lee L Age related macular degeneration - new developments in treatment Aust Fam Physician 2007 36 359 361 17492074

[B3] Chan CC Ross RJ Shen D Ding X Majumdar Z Bojanowski CM Zhou M Salem N Jr Bonner R Tuo J Ccl2/Cx3cr1-deficient mice: an animal model for age-related macular degeneration Ophthalmic Res 2008 40 124 128 10.1159/000119862 18421225 PMC2409067

[B4] Tuo J Bojanowski CM Zhou M Shen D Ross RJ Rosenberg KI Cameron DJ Yin C Kowalak JA Zhuang Z Zhang Z Chan CC Murine ccl2/cx3cr1 deficiency results in retinal lesions mimicking human age-related macular degeneration IOVS 2007 48 3827 3836 10.1167/iovs.07-0051 PMC2048751 17652758

[B5] Zhou Y Sheets KG Knott EJ Regan CE Jr Tuo J Chan CC Gordon WC Bazan NG Cellular and 3D optical coherence tomography assessment during the initiation and progression of retinal degeneration in the Ccl2/Cx3cr1-deficient mouse Exp Eye Res 2011 93 5 636 648 10.1016/j.exer.2011.07.017 21854772 PMC3221782

[B6] Tuo J Ross RJ Herzlich AA Shen D Ding X Zhou M Coon SL Hussein N Salem N Jr Chan CC A high omega-3 fatty acid diet reduces retinal lesions in a murine model of macular degeneration Am J Pathol 2009 175 799 807 10.2353/ajpath.2009.090089 19608872 PMC2716974

[B7] Tuo J Pang JJ Cao X Shen D Zhang J Scaria A Wadsworth SC Pechan P Boye SL Hauswirth WW Chan CC AAV5-mediated sFLT01 gene therapy arrests retinal lesions in Ccl2(-/-)/Cx3cr1(-/-) mice Neurobiol Aging 2011 33 2 433 e1-10 21397984 10.1016/j.neurobiolaging.2011.01.009 PMC3136657

[B8] Mehalow AK Kameya S Smith RS Hawes NL Denegre JM Young JA Bechtold L Haider NB Tepass U Heckenlively JR Chang B Naggert JK Nishina PM CRB1 is essential for external limiting membrane integrity and photoreceptor morphogenesis in the mammalian retina Hum Mol Genet 2003 12 2179 2189 10.1093/hmg/ddg232 12915475

[B9] Cao X Liu M Tuo J Shen D Chan CC The effects of quercetin in cultured human RPE cells under oxidative stress and in Ccl2/Cx3cr1 double deficient mice Exp Eye Res 2010 91 15 25 10.1016/j.exer.2010.03.016 20361964 PMC2879439

[B10] Ding X Patel M Shen D Herzlich AA Cao X Villasmil R Klupsch K Tuo J Downward J Chan CC Enhanced HtrA2/Omi expression in oxidative injury to retinal pigment epithelial cells and murine models of neurodegeneration IOVS 2009 50 4957 4966 10.1167/iovs.09-3381 PMC2903625 19443712

[B11] Herzlich AA Ding X Shen D Ross RJ Tuo J Chan CC Peroxisome proliferator-activated receptor expression in murine models and humans with age-related macular degeneration Open Biol J 2009 2 141 148 10.2174/1874196700902010141 21152244 PMC2998287

[B12] Herzlich AA Tuo J Chan CC Peroxisome proliferator-activated receptor and age-related macular degeneration [Abstract] PPAR Res 2008 2008 389507 18288287 10.1155/2008/389507 PMC2234091

[B13] Ross RJ Zhou M Shen D Fariss RN Ding X Bojanowski CM Tuo J Chan CC Immunological protein expression profile in Ccl2/Cx3cr1 deficient mice with lesions similar to age-related macular degeneration Exp Eye Res 2008 86 675 683 10.1016/j.exer.2008.01.014 18308304 PMC2375389

[B14] Shen D Cao X Zhao L Tuo J Wong WT Chan CC Naloxone ameliorates retinal lesions in Ccl2/Cx3cr1 double-deficient mice via modulation of microglia IOVS 2011 52 2897 2904 10.1167/iovs.10-6114 PMC3109007 21245403

[B15] Shen D Wen R Tuo J Bojanowski CM Chan CC Exacerbation of retinal degeneration and choroidal neovascularization induced by subretinal injection of Matrigel in CCL2/MCP-1-deficient mice Ophthalmic Res 2006 38 71 73 10.1159/000090266 16352919 PMC1930147

[B16] Verma V Sauer T Chan CC Zhou M Zhang C Maminishkis A Shen D Tuo J Constancy of ERp29 expression in cultured retinal pigment epithelial cells in the Ccl2/Cx3cr1 deficient mouse model of age-related macular degeneration Curr Eye Res 2008 33 701 707 10.1080/02713680802236185 18696346 PMC2535806

[B17] Cao X Shen D Patel MM Tuo J Johnson TM Olsen TW Chan CC Macrophage polarization in the maculae of age-related macular degeneration: a pilot study Pathol Int 2011 61 528 535 10.1111/j.1440-1827.2011.02695.x 21884302 PMC3292787

[B18] Bartosh TJ Ylostalo JH Mohammadipoor A Bazhanov N Coble K Claypool K Lee RH Choi H Prockop DJ Aggregation of human mesenchymal stromal cells (MSCs) into 3D spheroids enhances their antiinflammatory properties Proc Natl Acad Sci USA 2010 107 13724 13729 10.1073/pnas.1008117107 20643923 PMC2922230

[B19] Lee RH Pulin AA Seo MJ Kota DJ Ylostalo J Larson BL Semprun-Prieto L Delafontaine P Prockop DJ Intravenous hMSCs improve myocardial infarction in mice because cells embolized in lung are activated to secrete the anti-inflammatory protein TSG-6 Cell Stem Cell 2009 5 54 63 10.1016/j.stem.2009.05.003 19570514 PMC4154377

[B20] Milner CM Day AJ TSG-6: a multifunctional protein associated with inflammation J Cell Sci 2003 116 1863 1873 10.1242/jcs.00407 12692188

[B21] Milner CM Higman VA Day AJ TSG-6: a pluripotent inflammatory mediator? Biochem Soc Trans 2006 34 446 450 10.1042/BST0340446 16709183

[B22] Oh JY Roddy GW Choi H Lee RH Ylostalo JH Rosa RH Jr Prockop DJ Anti-inflammatory protein TSG-6 reduces inflammatory damage to the cornea following chemical and mechanical injury Proc Natl Acad Sci USA 2010 107 16875 16880 10.1073/pnas.1012451107 20837529 PMC2947923

[B23] Wisniewski HG Vilcek J TSG-6: an IL-1/TNF-inducible protein with anti-inflammatory activity Cytokine Growth Factor Rev 1997 8 143 156 10.1016/S1359-6101(97)00008-7 9244409

[B24] Paques M Guyomard JL Simonutti M Roux MJ Picaud S LeGargasson JF Sahel JA Panretinal, high-resolution color photography of the mouse fundus IOVS 2007 48 2769 2774 10.1167/iovs.06-1099 17525211

[B25] Ben-Shabat S Parish CA Hashimoto M Liu J Nakanishi K Sparrow JR Fluorescent pigments of the retinal pigment epithelium and age-related macular degeneration Bioorg Med Chem Lett 2001 11 1533 1540 10.1016/S0960-894X(01)00314-6 11412975

[B26] Karan G Lillo C Yang Z Cameron DJ Locke KG Zhao Y Thirumalaichary S Li C Birch DG Vollmer-Snarr HR Williams DS Zhang K Lipofuscin accumulation, abnormal electrophysiology, and photoreceptor degeneration in mutant ELOVL4 transgenic mice: a model for macular degeneration Proc Natl Acad Sci USA 2005 102 4164 4169 10.1073/pnas.0407698102 15749821 PMC554798

[B27] Parish CA Hashimoto M Nakanishi K Dillon J Sparrow J Isolation and one-step preparation of A2E and iso-A2E, fluorophores from human retinal pigment epithelium Proc Natl Acad Sci USA 1998 95 14609 14613 10.1073/pnas.95.25.14609 9843937 PMC24497

[B28] Wisniewski HG Naime D Hua JC Vilcek J Cronstein BN TSG-6, a glycoprotein associated with arthritis, and its ligand hyaluronan exert opposite effects in a murine model of inflammation Pflugers Arch 1996 431 R225 R226 10.1007/BF02346350 8739346

[B29] Choi H Lee RH Bazhanov N Oh JY Prockop DJ Anti-inflammatory protein TSG-6 secreted by activated MSCs attenuates zymosan-induced mouse peritonitis by decreasing TLR2/NF-kappaB signaling in resident macrophages Blood 2011 118 330 338 10.1182/blood-2010-12-327353 21551236 PMC3138686

[B30] Wisniewski HG Hua JC Poppers DM Naime D Vilcek J Cronstein BN TNF/IL-1-inducible protein TSG-6 potentiates plasmin inhibition by inter-alpha-inhibitor and exerts a strong anti-inflammatory effect *in vivo* J Immunol 1996 156 1609 1615 8568267

[B31] Sparrow JR Boulton M RPE lipofuscin and its role in retinal pathobiology Exp Eye Res 2005 80 595 606 10.1016/j.exer.2005.01.007 15862166

[B32] Caspi R Autoimmunity in the immune privileged eye: pathogenic and regulatory T cells Immunol Res 2008 42 41 50 10.1007/s12026-008-8031-3 18629448 PMC2756228

[B33] Kijlstra A La Heij E Hendrikse F Immunological factors in the pathogenesis and treatment of age-related macular degeneration Ocul Immunol Inflamm 2005 13 3 11 10.1080/09273940590909185 15804763

[B34] Morohoshi K Goodwin AM Ohbayashi M Ono SJ Autoimmunity in retinal degeneration: autoimmune retinopathy and age-related macular degeneration J Autoimmun 2009 33 247 254 10.1016/j.jaut.2009.09.003 19846275

[B35] Nussenblatt RB Ferris F Age-related macular degeneration and the immune response: implications for therapy Am J Ophthalmol 2007 144 618 626 10.1016/j.ajo.2007.06.025 17698021 PMC2744410

[B36] Umeda S Suzuki MT Okamoto H Ono F Mizota A Terao K Yoshikawa Y Tanaka Y Iwata T Molecular composition of drusen and possible involvement of anti-retinal autoimmunity in two different forms of macular degeneration in cynomolgus monkey (*Macaca fascicularis*) FASEB J 2005 19 12 1683 1685 16099945 10.1096/fj.04-3525fje

[B37] Liu B Wei L Meyerle C Tuo J Sen HN Li Z Chakrabarty S Agron E Chan CC Klein ML Chew E Ferris F Nussenblatt RB Complement component C5a promotes expression of IL-22 and IL-17 from human T cells and its implication in age-related macular degeneration J Transl Med 2011 9 1 12 21762495 10.1186/1479-5876-9-111 PMC3154861

[B38] Kehlen A Pachnio A Thiele K Langner J Gene expression induced by interleukin-17 in fibroblast-like synoviocytes of patients with rheumatoid arthritis: upregulation of hyaluronan-binding protein TSG-6 Arthritis Res Ther 2003 5 R186 R192 10.1186/ar762 12823853 PMC165059

[B39] Fries E Kaczmarczyk A Inter-alpha-inhibitor, hyaluronan and inflammation Acta Biochim Pol 2003 50 735 742 14515153

[B40] Tanigawa S Aida Y Kawato T Honda K Nakayama G Motohashi M Suzuki N Ochiai K Matsumura H Maeno M Interleukin-17 F affects cartilage matrix turnover by increasing the expression of collagenases and stromelysin-1 and by decreasing the expression of their inhibitors and extracellular matrix components in chondrocytes Cytokine 2011 56 2 376 386 10.1016/j.cyto.2011.08.015 21885294

[B41] Kumar A El-Osta A Hussain AA Marshall J Increased sequestration of matrix metalloproteinases in ageing human Bruch's membrane: implications for ECM turnover IOVS 2010 51 2664 2670 10.1167/iovs.09-4195 20042661

