# Supplementary material for: Anti-inflammatory recombinant TSG-6 stabilizes the progression of focal retinal degeneration in a murine model
Source: J Neuroinflammation. 2012 Mar 27;9:59. doi: 10.1186/1742-2094-9-59 (PMC3359240; doi:10.1186/1742-2094-9-59)
Supplement: Additional file 1 — Table S1. The list of mouse immune response genes. [file 1742-2094-9-59-S1.DOC]

Table S1: The list of mouse immune response genes
